# Heating Power of the Sun

**Published:** 1844-06-29

**Authors:** 


### HEATING POWER OF THE SUN.

Few of those who have not turned their minds to the particular study of HEAT, have estimated at its full amount the calorific power of the sun. We seldom, in these latitudes, find bodies exposed to the direct rays of the sun to attain a higher temperature than is indicated by about 120° Fah. But the celebrated M. de Saussure, by means of a little box constructed of wood, and lined with charred cork, obtained a temperature of 221° Fah., the temperature of the external air being at that time 55°; and Professor Robison, in the cold climate of Edinburgh, by means of a somewhat similar apparatus, frequently obtained an indication of 230°, and once, circumstances being favourable, of 237° Fah. The sun's rays, therefore, not *concentrated* in any way, but simply *accumulated*, have a temperature much above that of boiling water, in the 52d parallel of northern latitude. *Ibid.*

